# House-to-House Operating1Read at the twelfth annual meeting of the American Association of Obstetricians and Gynecologists, held at Indianapolis, September 19-21, 1899.

**Published:** 1899-11

**Authors:** Edwin Ricketts

**Affiliations:** Cincinnati, O.


### HOUSE-TO-HOUSE OPERATING.[1]

By EDWIN RICKETTS, M. D., of Cincinnati, O.

THE skilled abdominal and gynecological surgeon of today is a product of surgical evolution, to which no man ever gave such an impetus as the great and lamented Mr. Lawson Tait. His lines in surgical work were so simple, so thorough, so rapid, and withal so exacting on himself, assistants, nurses and patients, for pushing rapidly the case to a successful issue, or to the best possible result, that we should never lose sight of them, lest we forget that simplicity, rapidity, rigid cleanliness, and thoroughness are to be our key-note of success.

Mr. Tait began his work in his private house, without trained nurses, and even then had no unusual death-rate; but he gradually lowered the same markedly after training his assistants and nurses after his own fashion. After all of this experience he resorted to house-to-house operating, a most fitting close of his life's work in surgical diseases of women, which is food for sober thought, lest we are tempted to go off at a tangent by falling in line with the fad of the day—that of chemical antiseptics, as is only too often attempted to be practised in many of our hospitals.

The fresh air of country homes with their hygienic evolutions are still things paramount, that come to our aid in doing house-to-house surgery. While I am ready to acknowledge that the surgeon in our line of work, as in others, is improving, yet I stand ready to make the same claim for the general practitioner, in his ability to attend to the after-treatment, assisted by the nurse, aided by the telephone, telegraph and by rapid transit if the surgeon is needed. There is a mental dread of going into an institution which in many patients brings

---

1. Read at the twelfth annual meeting of the American Association of Obstetricians and Gynecologists, held at Indianapolis, September 19-21, 1899.

on, only too often, post-operative disasters that are undeniably eliminated by operating in their own homes; there is a love of home developed under just such trying circumstances to these patients, that should not be underestimated. How often do we see patients expressing a desire, after being operated upon in a hospital, either public or private, for something seemingly trivial from home, and how matters are helped if the request is granted and fulfilled.

Iron bedsteads, with other improved household furniture, are rapidly gaining access to many of the rural houses as permanencies, and yet they are not an absolute necessity for a patient that has to undergo a surgical operation. I beg to call your attention to the fact that the late Dr. Alexander Dunlap did 400 ovariotomies with a death-rate of but 15 per cent., never putting one of his patients on an iron bed; he never saw our modern operating tables with their multiplicity of contrivances for the greater part of his work was of the pioneer kind.

The numerous operating tables, giving all planes, from standing a patient on his head to the reverse position, found in many "diving tank" operating rooms, are not superior in obtaining results, to those of the kitchen table found in any house of the land; nor is the showy cleaning of the patient ever to be countenanced—especially while under anesthesia; such work should be done most thoroughly and most carefully before anesthesia is begun. The robing of operator, assistants and nurses in rubber suits and fishing boots, while the audience composing the "outer circle" is looked upon as being composed of persons teeming with dangerous and detestable microbes, is truly degrading to our art; it lowers the dignity and usefulness of our profession and is anything but a scientific show. This "outer-circle" soon makes up its mind—and justly, too,—that surely it is not so dirty, for the infected cases still follow notwithstanding the great hue and cry of these would be "holier than thou" surgeons.

Treves has put it well in saying that "such is more allied to the fervent idolatrous ritual brought down to the level of popular performance." The modern, smoothfaced, rubber-of-mail surgeon, with rubber gloves, face mask, boots, hat and coat, is truly a sight to behold, but not one for emulation, either from a scientific or a practical standpoint. He is a walking, self-instigated Turkish bath, and how the dirt with the epithelial microbes—anal and possibly urethral and vaginal as well—must pour down, mixed with perspiration, into the rubber wells of boots; this act of nature perspiring is certainly not a cleanly process, but it goes on right in the midst of operating, and is promoted and hastened by the wearing of the outfit above described.

My house-to-house work is done on the kitchen or dining-table in each house, properly arranged by the nurse sent ahead the day before I operate. The doctors invited are not asked if they had taken a bath; they were found to be gentlemen, which is sufficient for me; they come into the room when we are ready for them, look on, touch nothing in any way connected with the operation, and retire as soon as the operation is completed. Frequently some of the relatives desire to witness the operation and when it is possible one gains by allowing them to do so.

I take no steriliser to make a display in cleaning instruments or dressings; no baked glass jars for holding dressings—simply the original package—no foot giggers to cause the hot and cold water to flow; no glass-topped operating table; no dressing forceps, save nature's own, well cleaned before using them; no instruments resting in hot water, or in antiseptic solution; no blue, red or white water impregnated with chemicals to produce necrotic tissue; but after washing the abdomen thoroughly with soap and water, then likewise our hands, we applied freely 98 per cent. alcohol, and then began our work, using towels that had been dried in the sun and ironed by the help at hand.

My results have been all that the most fastidious could desire and of the houses of different construction that I have operated in, the old log house stands supreme for cleanliness much to the credit of its occupants, and which is all that need be desired.

Richelot, in a recent article On the relative value of antiseptics and improved technique, before the International Gynecological Congress says, "that the very best antiseptic does not retain its value for clinical purposes, and that laboratory prognoses were not always to be relied upon. It was found that the use of antiseptics was not only inefficient, but at times dangerous, hence it gradually became more or less discredited, whilst sterilisation by heat has been daily gaining favor." He further says, "the best results are obtained by the surgeon who knows how to use his hands and his common sense, and that methods continue to become more and more simplified, and in that way lies progress."

Bumm, on the same subject, says, "the absolute reliance on the protective power of antiseptics has been a good deal diminished by exact investigations, as it has been proved by a whole series of experiments and observations, that the elimination of all microorganisms during the operation has not been attained; there is no way to remove, with certitude, all microörganisms from the hands; it is therefore illusory to think that either asepsis or antisepsis can bring about a sterile condition of the wound; in spite of the proved presence of microörganisms, most wounds heal without suppuration and fever. In addition to this, short operations do not so much tax the resisting power of the wounded tissues as well as the entire organism. To expose the peritoneal cavity during a long time has a well known bad influence on the heart, the intestines, and the serosa. Asepsis has delivered us of the dangerous application of too much concentrated disinfectants. Antiseptics and improved technique have to go hand in hand—the dryer the wound the better are the chances for primary healing."

The last statement I want to lay stress upon as being a surgical fact recognised for centuries. The tendency of medicine and surgery is too prone to claim that many individual views are new when the author has been dead for years. Let us never fail to honor the memory, when possible, of those to whom honor is due. Thorough operating, under rules of simple cleanliness, will continue to produce more and better results than is possible with the strictest rules of antisepsis; slow, tedious operating, carried out with hesitancy, is always to be condemned.

House-to-house operating means house-to-house isolation and for this the room-to-room risks of infection in a hospital are lessened to a minimum degree. Surgical cleanliness is surgical godliness, and every time we forget this we add to our death-rate. Chemical perfumes of varied kinds will never displace soap and hot water judiciously applied in surgery.

The average rural home location is not bad, and with scrubbing-brush, water and soap, it can be continually made to shine out for the demonstration of surgical truths, that are Gibraltars to us; they are the indwellings of the people, who have long since learned to love and cherish them in sickness as well as in health. Our best surgery can be given them here, and our reward is to be the lowest death-rate possible.

415 BROADWAY.

---

THE Woman's Sanitary League of Pennsylvania has been organised at Philadelphia, with the objects of advancing sanitary science, promoting public health and good government, and forming country and local auxilaries to extend the beneficent work.—*Medical Age.*

